# Exploring awareness, perceptions, and practices relating to nutritional status and low muscle mass in patients with ovarian cancer

**DOI:** 10.1007/s00520-025-09739-5

**Published:** 2025-07-21

**Authors:** Sarah Benna-Doyle, Nicole Kiss, Erin Laing, Jenelle Loeliger, Brenton J. Baguley

**Affiliations:** 1 Institute for Physical Activity and Nutrition, Deakin University, Geelong, VIC Australia; 2 School of Exercise and Nutrition Sciences, Deakin University, Burwood, VIC Australia; 3 Nutrition and Speech Pathology Department, Peter MacCallum Cancer Centre, Melbourne, VIC Australia; 4 Sir Peter MacCallum Department of Oncology, The University of Melbourne, Melbourne, VIC Australia

**Keywords:** Ovarian cancer, Malnutrition, Screening, Nutrition, Sarcopenia

## Abstract

**Purpose:**

Women with ovarian cancer are at high risk of malnutrition and muscle loss due to advanced-stage diagnosis and treatment toxicities. Evidence-based guidelines recommend screening for malnutrition and sarcopenia to prevent associated adverse consequences, including reduced survival. This study aimed to describe awareness and perceptions of nutrition-related issues and practices in ovarian cancer among Australian healthcare professionals.

**Methods:**

A national survey was completed between November 2023 and March 2024. The 24-item survey evaluated awareness and perceptions of nutrition-related issues, screening and referral practices for malnutrition and sarcopenia at specific timepoints (from diagnosis, during, and/or post-treatment) and barriers to nutrition care in ovarian cancer.

**Results:**

Professionals (*n* = 57) were predominantly nurses (39%), dietitians (23%), or surgeons (19%). The most reported nutrition-related issues at diagnosis were weight loss (67%), overweight/obesity (54%), and sarcopenia (44%). During treatment, weight loss (70%) and sarcopenia (65%) were prominent, while post-treatment, weight gain (46%) and sarcopenia (39%) were most reported. The perceived clinical importance of malnutrition and sarcopenia varied according to professional discipline. Professionals identified chemotherapy as the treatment with the highest nutrition risk (97%), and 75% reported observing self-initiated dietary changes during treatment, yet 18% indicated their health services did not screen for nutrition risk, and 58% did not screen for sarcopenia. Key barriers were lack of established processes for sarcopenia screening (75%), limited dietetic services (60%), and lack of specific referral pathways (58%).

**Conclusion:**

The perceived importance of malnutrition and sarcopenia in ovarian cancer varies according to professional discipline despite robust evidence of the importance to clinical outcomes.

**Supplementary Information:**

The online version contains supplementary material available at 10.1007/s00520-025-09739-5.

## Introduction

Ovarian cancer is the most fatal gynaecological cancer both globally and within Australia [1, 2]. Most women are diagnosed at an advanced stage (III–IV), and less than half will survive 5 years [3]. A diagnosis of advanced ovarian cancer often requires invasive treatment, including maximal cytoreductive surgery with chemotherapy before and/or after surgery [4]. The ability to endure intensive treatment is highly dependent upon nutritional status, muscle mass, and cardiorespiratory fitness [5]. Women with ovarian cancer are more likely to be malnourished at the time of diagnosis than women with other gynaecological cancers due to late-stage presentation with gastrointestinal involvement, causing digestive symptoms and impaired nutrient absorption [6, 7]. Up to 40% of women have low muscle mass at the time of diagnosis [8], and for most women, treatment will increase the risk of nutritional decline and muscle loss [6]. Malnutrition and muscle loss in patients with ovarian cancer are associated with poorer surgical outcomes [9, 10], increased hospitalisation [11, 12], and worse overall survival [9, 13, 14].

Accelerated nutritional decline in people with cancer is multifaceted and attributable to a combination of tumour and treatment-related factors [15]. Specifically in advanced ovarian cancer, the pro-catabolic and pro-inflammatory effects of cancer itself, the location of the tumour, and cancer treatment increase nutritional risk [6, 16]. Muscle loss is a key component of cancer-related malnutrition [17], and similarly to malnutrition, cancer-related muscle loss is multifaceted [18, 19]. Whilst malnutrition and muscle loss have overlapping aetiologies, these conditions can occur independently or concomitantly [17, 20]. While traditionally, unintentional weight loss has been considered a hallmark sign of cancer-related malnutrition, malnutrition and low muscle mass occur in all body mass index (BMI) classifications [21]. Concurrently, the prevalence of overweight and obesity in patients with cancer continues to increase [22]. Therefore, these conditions may be overlooked in patients who are overweight and obese and not prioritised for dietetic intervention [21]. Given the serious adverse consequences of both malnutrition and muscle loss in patients with ovarian cancer, proactive processes with multidisciplinary coordination to accurately identify risk and intervene before these conditions progress are instrumental to achieving better outcomes [22].

National and international evidence-based guidelines and position statements strongly recommend screening at diagnosis with repeated monitoring to identify nutrition risk for early dietetic referral [17, 20, 23]. However, research consistently shows poor integration of recommendations into clinical practice across multiple healthcare settings [24]. Malnutrition and sarcopenia screening in ovarian cancer, where the risk is high, is relatively unknown. A retrospective analysis showed screening and overall referral to dietetics services for women with gynaecological cancer undergoing pelvic radiotherapy was poor (48% referred) despite high rates of weight loss and nutrition-impact symptoms (NIS) during and post-treatment [25]. Research in ovarian cancer specifically has focused on the provision of dietetic services after treatment to manage latent NIS and monitor nutritional status [26, 27]. Referral pathways and access to dietetic services for patients with ovarian cancer from the time of diagnosis and during treatment are yet to be investigated. Therefore, the primary aims of this survey were to (1) describe multidisciplinary healthcare professional (HCP) awareness of nutrition-related issues, when they occur, and perceptions of clinical importance to the treatment and management of patients with ovarian cancer, and (2) investigate malnutrition and sarcopenia screening and referral practices among health services in Australia. In addition, this study aimed to identify barriers to providing nutrition-related services for patients with ovarian cancer.

## Methods

### Study design and setting

A national cross-sectional survey of Australian multidisciplinary HCPs between November 2023 and March 2024. This online survey was developed and reported according to the CHERRIES (Checklist for Reporting Results of Internet E-Surveys) guidelines [28]. Eligible participants were ≥ 18 years, from any multidisciplinary profession, and currently, or within the last five years, caring for patients with ovarian cancer in the Australian healthcare setting. Ethics approval was obtained from the Deakin University Health Ethics Advisory Group (HEAG-H 145_2023).

The 24-item online open survey was created in the Qualtrics^XM^ programme (Qualtrics, Provo, UT, USA). The survey was anonymous, and each participant received a unique link to access the survey. To maintain data integrity, the platform tracked IP addresses to identify and prevent multiple submissions from the same participant while remaining anonymous. Potentially eligible participants were recruited through voluntary and snowballing sampling via email through professional networks of the research team, distribution lists of professional cancer organisations (Clinical Oncology Society of Australia) or ovarian cancer-specific professional organisations (Ovarian Cancer Australia [OCA], Australian Society of Gynaecological Oncologists, Australia New Zealand Gynaecological Oncology Group). Social media accounts on X operated by Deakin University and OCA were also used for recruitment. Reminder emails were sent to participants two weeks after the initial email invitation for those associated with professional cancer organisations. Participation was voluntary, and eligible participants provided informed consent through a participant information sheet embedded in the Qualtrics link prior to commencing the survey.

### Survey instrument

The survey was purposefully designed following review of the published literature and iteratively developed by members of the research team with expertise in nutrition and cancer (Online Resource 1). The survey captured basic demographic information, including professional discipline, state/territory of practice, practice setting (e.g. public, private), self-reported geographic region (metropolitan, rural/regional), proportion of time spent working in ovarian cancer, and years of experience.

The 24-item questionnaire was designed to capture five key areas: (1) awareness and perspectives of nutrition-related issues at specific time points (at diagnosis, during, and/or post-treatment) and the treatments most likely to lead to nutrition-related issues, (2) common symptoms reported by patients and when these are most likely to occur (at diagnosis, after surgery, during or after chemotherapy, radiotherapy, immunotherapy), (3) perspectives of the clinical importance of nutritional status and sarcopenia in the context of overall treatment, (4) screening and referral practices for nutrition risk and sarcopenia and when screening and referral occurs (at diagnosis, before/after surgery, before/during/after chemotherapy, radiotherapy, immunotherapy, on admission, and/or attendance to outpatient), and (5) barriers to providing nutrition services for patients with ovarian cancer.

Questions on perceptions of nutrition-related issues, symptoms, and the clinical importance of nutritional status and sarcopenia were asked at an individual level. Questions on screening and referral practices and barriers to nutrition services were asked at a health service level. Questions on frequency or level of agreement were assessed using 5-point Likert scales (from “always” to “never” and “strongly disagree” to “strongly agree”). Questions on perceptions of importance and likeliness were assessed on a 7-point Likert scale (0 = extremely unlikely/not at all important to 7 = extremely likely/extremely important). Potential barriers to providing nutrition services were informed by earlier research among cancer clinicians [29]. The level of agreement with each barrier was assessed on a 5-point Likert scale (as above).

Where possible, all questions included an “unsure”, “not applicable”, or “other” response with a free text option. Skip logic was applied to follow-up questions regarding screening (e.g. timing, who is responsible) when participants selected “unsure” or “to my knowledge, my health service does not screen for nutrition risk/sarcopenia”, directing them to the next section. Participants were able to review and change their answers when completing the survey. Using previously reported methodology [30, 31], the survey was tested for face validity by four HCPs (two dietitians, one medical oncologist and a nurse consultant). The four HCPs provided written feedback on readability and clarity of the survey instrument after completing a test of the online survey.

### Statistical analysis

Data analysis was performed using *R* (Version 4), with only completed questionnaires with no missing data included. Descriptive statistics, including counts and percentages, were used to summarise participant characteristics and survey responses. Likert scale questions were plotted using the *Likert* R package (Bryer, J., Speerschneider, K.) to represent percentage distributions across scale categories. Median and interquartile ranges [IQR] were used to summarise the 7-point Likert scale responses regarding symptoms perceived as most likely to lead to nutrition-related issues and perceptions of the importance of nutritional status and sarcopenia. To explore discipline-specific responses, participants were grouped according to professional discipline and included (1) dietitian, (2) nurse, (3) medical professional (medical oncologists and surgeons), and (4) other (physiotherapist, exercise physiologist, and all “other” responses). Responses to the 7-point Likert scale on importance were also regrouped by responses of 6 or 7 to represent “Important” and categorically analysed by professional discipline.

Responses to the 5-point Likert scales assessing symptoms most reported by patients and barriers to nutrition screening and referrals were recoded into three groupings. Frequency was regrouped as (1) “mostly-always” (representing “always” and “most of the time”), (2) “about half the time”, and (3) “mostly-never” (representing “sometimes” and “never”). The level of agreement was regrouped as (1) “disagree” (representing “strongly disagree” and “somewhat disagree”, (2) “neither agree nor disagree”, and (3) “agree” (representing “somewhat agree” and “strongly agree”).

## Results

A total of 57 HCPs from a range of disciplines participated (Table 1). The majority were nurses (*n* = 22, 39%), dietitians (*n* = 13, 23%), and surgeons (*n* = 11, 19%); worked in the public hospital setting (*n* = 37, 65%) in a metropolitan location (*n* = 38, 67%); and spent < 50% (*n* = 41, 72%) of their caseload working with patients with ovarian cancer. Most participants (42%) had worked in ovarian cancer for 6–10 years, and all regions of Australia were represented.

**Table 1:** Characteristics of survey participants

Participants	*N* = 57
**Professional discipline**	
Medical professional	
Surgeon	11(19%)
Medical oncologist	4 (7%)
Allied health professional	
Nurse	22 (39%)
Dietitian	13 (23%)
Exercise physiologist	4 (7%)
Physiotherapist	1 (2%)
Other health professional^a^	2 (4%)
**Practice setting**^**b**^	
Public hospital	37 (65%)
Private hospital	2 (3%)
Public and private hospital	5 (9%)
Private practice/community healthcare	8 (14%%)
Other	7 (12%)
**Geographic location**	
Metropolitan	38 (67%)
Rural/regional	11(19%)
Both	8 (14%)
**Years of experience in ovarian cancer**	
1–5 years	14 (25%)
6–10 years	24 (42%)
11–20 years	6 (11%)
> 20 years	13 (23%)
**Clinical time spent working in ovarian cancer**	
≤ 5%	8 (14%)
> 5% but ≤ 25%	13 (23%)
> 25% but ≤ 50%	20 (35%)
> 50% but ≤ 75%	8 (14%)
> 75% (almost all caseload)	8 (14%)

^a^Other health professionals included a naturopath and an allied health assistant

^b^Multiple responses allowed for practice location

### Nutrition-related issues identified in ovarian cancer

**Fig. 1:**
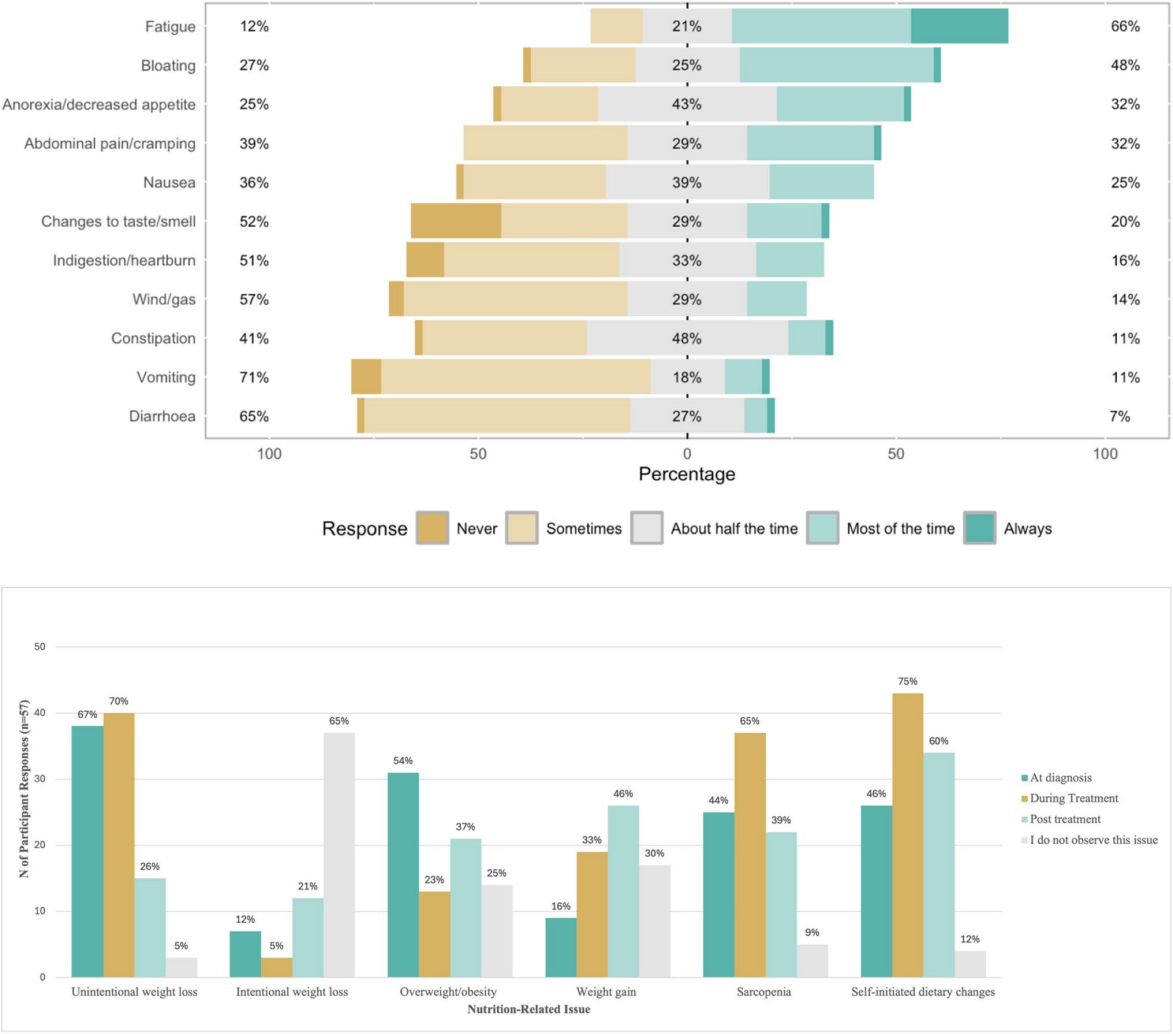
**a** Most common nutrition-related symptoms at any time point in patients with ovarian cancer, as reported by healthcare professionals. Percentages represent the proportion of responses to “never” and “sometimes” combined, “about half the time”, and “most of the time” and “always” combined. **b** Most observed nutrition-related issues by time point in patients with ovarian cancer, as reported by healthcare professionals

The most common nutrition-related symptoms, at any time point, reported by HCPs were fatigue (*n* = 37, 66%), bloating (*n* = 27, 48%), anorexia/decreased appetite (*n* = 18, 32%), and abdominal pain/cramping (*n* = 18, 32%) (Fig. 1a). The median [IQR] for symptoms HCPs identified as most likely to lead to nutrition-related issues were anorexia (6.0 [5-7]), early satiety (6.0 [5-7]), and nausea (6.0 [4-7]) were consistent according to professional discipline.

When asked when patients were most likely to experience the greatest nutrition-related symptom burden, HCPs indicated at diagnosis (*n* = 32, 56%), during chemotherapy (*n* = 30, 53%), and after radiotherapy (*n* = 13, 23%). Results for all time points are reported in Online Resource 2. Nearly all HCPs (*n* = 55, 97%) identified chemotherapy as the treatment with the greatest risk of nutritional decline, followed by surgery (*n* = 34, 60%) and immunotherapy (*n* = 18, 32%). Unintentional weight loss, overweight and obesity, sarcopenia, and self-initiated dietary changes were the most observed nutrition-related issues across various time points when caring for patients with ovarian cancer (Fig. 1b). At diagnosis, unintentional weight loss (*n* = 38, 67%), overweight/obesity (*n* = 31, 54%), and sarcopenia (*n* = 25, 44%) were prominent. During treatment, unintentional weight loss (*n* = 40, 70%) and sarcopenia (n = 37, 65%) were most prominent. Post-treatment, weight gain (*n* = 26, 46%), sarcopenia (*n* = 22, 39%), and overweight/obesity (*n* = 21, 37%) were the most commonly observed nutrition-related issues. A high proportion of HCPs also reported patients were self-initiating dietary changes at the time of diagnosis (*n* = 26, 46%), during treatment (*n* = 43, 75%), and post-treatment (*n* = 34, 60%). Most observed nutrition-related issues, including self-directed dietary changes with corresponding time points, are shown in Fig. 1b.

### Nutritional status, sarcopenia, and screening practices

Overall, both nutritional status and sarcopenia were perceived as clinically important to the treatment and management of patients with ovarian cancer. The median [IQR] response from HCPs for the importance of nutritional status (score 0–7) was 7.0 [6-7], and for sarcopenia, the median response was 6.0 [5-7]. When responses were regrouped to represent “important” and analysed by professional discipline, it was revealed that 100% of dietitians, 91% of nurses, 67% of medical professionals, and 86% of “other” HCPs considered nutritional status “important” (score 6–7). For sarcopenia, 100% of dietitians and “other” health professionals, 68% of nurses, and 40% of medical professionals considered sarcopenia “important”.

#### Nutrition risk screening

Overall, 18% (*n* = 10) of HCPs reported nutrition risk screening did not occur or they were unsure if it occurred in their health service. Among participants who reported screening for nutrition risk, screening was perceived as a responsibility of the nurse (*n* = 35, 61%), dietitian (*n* = 30, 53%), and/or medical oncologist (*n* = 12, 21%). The most used screening tools were the Malnutrition Screening Tool (MST; *n* = 32, 68%), the Malnutrition Screening Tool for Cancer (MSTC; *n* = 6, 13%), and/or biochemical measures (e.g. albumin; *n* = 6, 13%). Thirteen participants (28%) were unsure of the screening tool used in their health service. Timing of screening varied, with admission (*n* = 15, 32%) before surgery (*n* = 14, 30%) and before chemotherapy (*n* = 12, 26%) the most reported time points. Results for all nutrition risk screening time points are reported in Online Resource 2.

#### Sarcopenia screening

The majority of participants (*n* = 33, 58%) reported their health service either did not screen for sarcopenia or they were unsure. Perceptions of responsibility varied across health services, with HCPs identifying dietitians (*n* = 16, 28%), nurses (*n* = 9, 16%), and others unsure who was responsible (*n* = 9, 16%). Among HCPs who reported screening for sarcopenia, BMI (*n* = 10, 42%) was most commonly used, followed by the SARC-F plus calf circumference (SARC-CalF) (*n* = 4, 17%) or a measure of muscle strength (*n* = 4, 17%) and/or physical function (*n* = 4, 17%), whilst 38% (*n* = 9) of participants were unsure what tool or method was used to screen for sarcopenia. Before surgery (*n* = 8, 33%), after surgery (*n* = 8, 33%), and at diagnosis (*n* = 7, 29%) were the most reported time points for screening. Results for all sarcopenia screening time points are reported in Online Resource 2.

#### Dietetic referral

Nearly all HCPs (*n* = 52, 91%) indicated their health services had dietetic services available to refer patients with ovarian cancer. The primary reasons for a dietetic referral were unintentional weight loss or malnutrition (*n* = 45, 87%), patient/carer request (*n* = 27, 52%), and symptom management (*n* = 23, 44%). The most common time points for dietetic referral were at diagnosis (*n* = 21, 40%), after surgery prior to adjuvant chemotherapy (*n* = 18, 35%), and during neoadjuvant (*n* = 16, 31%) or adjuvant chemotherapy (*n* = 15, 29%). Referral after treatment was the least reported time point compared with before or during treatment. Nine HCPs (17%) indicated referral occurred after neoadjuvant chemotherapy, four (7%) after adjuvant chemotherapy, and two (4%) after radiotherapy. Results for all referral time points are reported in Online Resource 2.

### Barriers

**Fig. 2:**
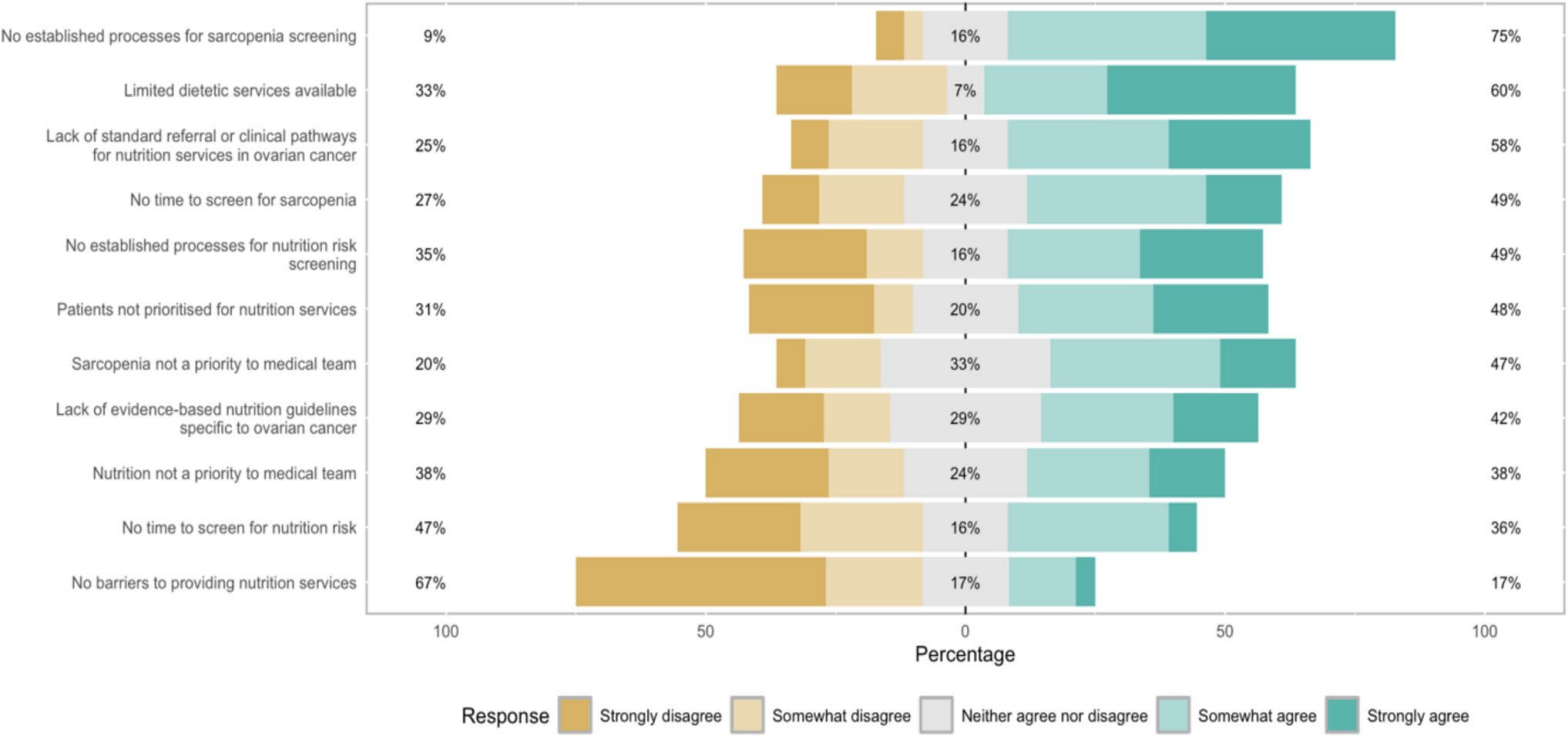
Perceived barriers to the provision of nutrition services in ovarian cancer. Percentages represent the proportion of responses to “strongly disagree” and “somewhat disagree” combined, “neither agree nor disagree”, and “somewhat agree” and “strongly agree” combined

The majority of HCPs (*n* = 36, 67%) indicated there were barriers to providing nutrition services to patients with ovarian cancer in their health service (Fig. 2). The top barriers included (1) no established process for sarcopenia screening (*n* = 41, 75%), (2) limited dietetic services available (*n* = 33, 60%), and (3) lack of standard referral pathways specific to ovarian cancer (*n* = 32, 58%) (Fig. 2).

## Discussion

To our knowledge, this study is the first to investigate HCP awareness and perceptions of nutrition-related issues, current practices, and barriers to providing nutrition care for patients with ovarian cancer in Australia. This survey revealed four important findings: (1) nutrition-related symptoms were reported at diagnosis as well as during treatment, while nutrition-related issues were observed across all time points; (2) screening practices, including the use of validated screening tools, were inconsistent, and most health services did not screen for sarcopenia; (3) perceptions of the clinical importance of nutritional status and sarcopenia vary according to professional discipline; and (4) a lack of established processes for sarcopenia screening, limited dietetic services, and lack of specific referral pathways were key barriers to providing nutrition-related services for patients with ovarian cancer. The findings from this sample of HCPs suggest that nutrition care practices for patients with ovarian cancer, a vulnerable patient group, currently do not align with best practice guidelines for a coordinated multidisciplinary approach to the identification and management of cancer-related malnutrition and sarcopenia [20, 23]. These challenges, however, are not unique to ovarian cancer. Lack of recognition for the impact of sarcopenia and malnutrition on clinical outcomes, resource limitations, and guideline integration at a systems level are pervasive challenges to providing nutrition care in oncology despite the evidence [20, 32].

The presence of NIS in patients with cancer is a key predictor of malnutrition risk [33], and the time point(s) for when symptoms are likely to occur aid in guiding proactive nutrition screening and referral practices [20, 34]. In this survey, HCPs identified fatigue, bloating, abdominal pain, and anorexia as the nutrition-related symptoms most reported, at any time point, by their patients with ovarian cancer. These symptoms are prognostically significant due to their impact on nutritional intake and ability to maintain protein and energy requirements [35]. Dietary intake is modifiable and an opportunity for intervention to preserve lean muscle mass and enhance treatment tolerance and recovery, when identified early [20]. In women with ovarian cancer, earlier research has shown that protein intake at or above guideline recommendations (> 1 g/kg/day) post-treatment has been associated with improved progression-free survival [36]. Thus, dietary intervention across all phases is an important opportunity to reduce morbidity and mortality [27]. In this study, HCPs also identified the time of diagnosis, during chemotherapy, and after radiotherapy as the time points with the greatest NIS burden, yet screening practices were inconsistent and varied. This might explain the high prevalence of self-initiated dietary changes HCPs also reported observing in their patients with ovarian cancer across all time points. Other research in ovarian cancer has reported on dietary changes post-treatment and found that although HCPs are the preferred source of information, women with unmet needs may turn to unreliable sources that are not evidence-based, which may increase risk of both malnutrition and muscle loss [37]. These results indicate that women are experiencing symptoms from the time of diagnosis that require nutrition intervention, underscoring the importance of early and repeated screening that considers the presence of NIS. However, patients with ovarian cancer are not traditionally viewed as at high risk of malnutrition in comparison to patients with head and neck or gastrointestinal cancers [38]. In this survey, patient/carer request was also a primary reason for making a dietetic referral. Earlier research in ovarian cancer has shown that after treatment, diet and nutrition-related information and referral were often dependent upon the patient explicitly indicating a need for support in the outpatient setting [27]. These findings also highlight a need to further explore patient experiences with care and access to nutrition services from diagnosis and throughout treatment.

According to HCPs, unintentional weight loss and sarcopenia were among the most observed issues both at the time of diagnosis and during treatment, while sarcopenia was also observed following treatment. Importantly, overweight and obesity were also commonly observed at diagnosis and post-treatment, with weight gain also among the most observed nutrition-related issues post-treatment. While unintentional weight loss is a traditional indicator of malnutrition in patients with cancer, a more contemporary understanding of body composition has shown that muscle loss may have greater clinical relevance than weight loss alone, and both malnutrition and muscle loss can occur irrespective of BMI and overt changes in weight [39–41]. Thus, in patients who are overweight or obese or in those who gain weight, nutritional decline and muscle loss may go undetected [39]. This is of particular importance for women with ovarian cancer who are primarily diagnosed at a menopausal age [42], characterised by changes in body composition, including muscle loss and changes in body fat distribution with or without changes in body weight [43]. The additional limitation of using BMI alone to assess body composition in ovarian cancer due to disease-related factors such as ascites is also well documented [10]. Consequently, our finding that the majority of respondents reported their health services did not screen for sarcopenia and conventional reliance on BMI suggests a high proportion of women with muscle loss across all time points are potentially going undetected. Additionally, it does not appear that obesity or sarcopenia commonly necessitate a dietetic referral. In this study, unintentional weight loss or malnutrition was the foremost reason for a dietetic referral, with referrals most commonly occurring at diagnosis and during treatment. In Australia, the Optimal Care Pathway for women with ovarian cancer outlines both obesity and malnutrition as key components of patient performance status and advises dietetic referral for management prior to treatment to improve clinical outcomes [44]. While post-treatment, in other gynaecological cancers, obesity is associated with increased risk of cancer recurrence and mortality [45], and should be a reason for dietetic referral and intervention.

Identifying risk of malnutrition and sarcopenia and referring for early nutritional management is not without challenge in the clinical setting [21]. Whilst evidence-based guidelines reiterate the need for a multidisciplinary approach [20, 21], this survey revealed differences in perceptions of clinical importance according to professional discipline. Two-thirds of medical professionals perceived nutritional status as important to the overall treatment and management of patients with ovarian cancer, and only 40% viewed sarcopenia as important. These findings differ from an earlier Australian survey among oncology clinicians, which found high awareness of the importance of these conditions irrespective of discipline [29]. It is worth noting that multiple factors influence malnutrition and sarcopenia awareness and practices across health services, which include, but are not limited to, the low prevalence of this cancer, lack of training or it being non-specific to ovarian cancer, and variations in the exposure to the multidisciplinary management of women with ovarian cancer [46]. The findings from our study suggest the clinical implications of both sarcopenia and malnutrition in patients with ovarian cancer are underestimated by medical professionals, and there is a need to raise awareness. HCPs also identified a lack of established processes for sarcopenia screening, limited dietetic services, and a lack of specific referral pathways specific to patients with ovarian cancer as key barriers to providing nutrition care. Whilst sarcopenia screening was not as common as malnutrition screening in this survey, previous research has shown screening for sarcopenia is feasible and acceptable within the inpatient setting across various cancer diagnoses [47]. Validated screening methods for sarcopenia are available (SARC-F, SARC-F with calf circumference) [20], although not commonly used by HCPs as reported in our survey. In this survey, BMI was the most used method to screen for sarcopenia, which may suggest low awareness of available validated tools. These findings highlight the need to improve multidisciplinary communication on the clinical implications of malnutrition and sarcopenia and the important role all healthcare professionals across the multidisciplinary team play in identification to facilitate early referral and intervention [20]. To support this, a clearly defined clinical pathway specific to nutrition care in patients with ovarian cancer would aid in streamlining consistent evidence-based screening practices at key time points to identify and prioritise patients at risk for appropriate assessment and intervention.

## Limitations

Whilst this study is the first to provide insight into the current landscape of nutrition care during treatment for patients with ovarian cancer in Australia, it also has some limitations. The recruitment strategy used in this study did not allow for the calculation of response rate. Therefore, it was not possible to determine if our sample was representative of multidisciplinary HCPs working in ovarian cancer across Australia. We acknowledge the small sample size and a lower response rate for medical oncologists compared with surgeons, nurses, and dietitians. Rural healthcare services, private practice, and community health services were also underrepresented. Additionally, participants with an interest in and prior knowledge of nutrition may have preferentially participated in this survey, contributing to selection bias that could have led to an overestimation of nutrition-related issues and skewed the findings regarding perceptions of importance. Lastly, this survey was unable to evaluate *all barriers and facilitators* to screening and referral practices across health services in Australia due to the dynamic nature of these processes at a services level and is an area for further exploration.

## Future directions

The results of this study highlight key areas for future research and steps to address HCP awareness of malnutrition and sarcopenia in patients with ovarian cancer to facilitate consistent, evidence-based screening and referral practices. First, to increase awareness of nutrition risk and shift perceptions of malnutrition and sarcopenia, the development and dissemination of nutrition educational programs specific to ovarian cancer should be a priority. This recommendation follows a recent survey finding 99% of oncology professional respondents were interested in advanced education on clinical nutrition topics [48]. An evidence-based clinical pathway specific for nutrition care in patients with ovarian cancer would aid in reducing variation in screening practices, improve alignment with clinical guidelines, and provide consistent, high-quality care. With regard to sarcopenia screening, additional implementation resources that include training on recommended screening tools may aid in raising awareness of the limitations of BMI as a metric for muscle loss [29]. Lastly, the high prevalence of self-initiated dietary changes observed in this survey highlights a need to understand the influence of NIS on dietary change at diagnosis and throughout treatment to identify unmet nutrition-related needs and explore the patient experience across all treatment modalities to inform patient-centred nutrition care. These findings also suggest an opportunity to engage and empower patients on the importance of nutrition and maintaining muscle mass during treatment and support self-advocacy. Although this study focused on ovarian cancer, the under-estimation of malnutrition and sarcopenia and poor integration of nutrition care recommendations in clinical practice are documented challenges in the oncology setting more broadly [20, 32]. While these recommendations are specific to the nutritional needs of patients with ovarian cancer, future research could explore if similar barriers exist and whether these approaches are generalisable to other cancer populations.

## Conclusion

Cancer-related malnutrition and sarcopenia represent modifiable prognostic factors that, when appropriately identified and managed, have the potential to improve treatment outcomes. However, their prognostic capability is reliant upon a coordinated multidisciplinary approach to identification and timely referral for appropriate intervention. This survey identified a need to raise awareness of the serious adverse consequences of malnutrition and muscle loss in patients with ovarian cancer, particularly among medical professionals. Tailored clinical pathways for nutrition care in ovarian cancer, integrated within existing multidisciplinary pathways, would support consistent evidence-based nutritional care in a population where both risk and consequence may be underestimated.

## Supplementary Information

Below is the link to the electronic supplementary material.

Supplementary file1 (DOCX 33 KB)

Supplementary file2 (DOCX 23 KB)

## Data Availability

The anonymous data collected from this survey is available from the corresponding author upon request following appropriate data share agreements.

## References

[1] Huang JU, Chan WI, Ngai CH, Lok VE, Zhang LI, Lucero-Prisno DO, 3rd, et al (2022) Worldwide burden, risk factors, and temporal trends of ovarian cancer: a global study. Cancers (Basel). 14(9) 10.3390/cancers14092230 10.3390/cancers14092230 PMC9102475 35565359

[2] Ovarian Cancer Research Foundation (2020) State of the Nation in Ovarian Cancer: Research Audit. https://evenicoocrf2019.blob.core.windows.net/assets/pages/OCRF_State%20of%20the%20Nation%20Report_FINAL%20%5B12%20August%202020%5D.pdf. Accessed 3 Jun 2025

[3] Cancer Australia (2025) Ovarian cancer statistics in Australia: Australian Government; 2025 [19 February 2025]. Available from: https://www.canceraustralia.gov.au/cancer-types/ovarian-cancer/ovarian-cancer-statistics-australia. Accessed 9 June 2025

[4] Armstrong DK, Alvarez RD, Bakkum-Gamez JN, Barroilhet L, Behbakht K, Berchuck A et al (2021) Ovarian cancer, Version 2.2020, NCCN clinical practice guidelines in oncology. J Natl Compr Canc Netw 19(2):191–226. 10.6004/jnccn.2021.0007 33545690 10.6004/jnccn.2021.0007

[5] Elsherbini NO, Carli FR (2022) Advocating for prehabilitation for patients undergoing gynecology-oncology surgery. Eur J Surg Oncol 48(9):1875–1881. 10.1016/j.ejso.2022.04.021 35534307 10.1016/j.ejso.2022.04.021

[6] Obermair AN, Simunovic MA, Isenring LI, Janda MO (2017) Nutrition interventions in patients with gynecological cancers requiring surgery. Gynecol Oncol 145(1):192–199. 10.1016/j.ygyno.2017.01.028 28173966 10.1016/j.ygyno.2017.01.028

[7] Benna-Doyle S, Baguley BJ, Laing E, Kiss N (2024) Nutritional interventions during treatment for ovarian cancer: a narrative review and recommendations for future research. Maturitas 183:107938. 10.1016/j.maturitas.2024.107938 38367367 10.1016/j.maturitas.2024.107938

[8] Tranoulis AN, Kwong FO, Lakhiani AA, Georgiou DI, Yap JA, Balega JA (2022) Prevalence of computed tomography-based sarcopenia and the prognostic value of skeletal muscle index and muscle attenuation amongst women with epithelial ovarian malignancy: a systematic review and meta-analysis. Eur J Surg Oncol 48(7):1441–1454. 10.1016/j.ejso.2022.02.024 35260290 10.1016/j.ejso.2022.02.024

[9] Nasser SA, Bilir ES, Derin XE, Richter RO, Grabowski JA, Ali PA et al (2024) Pre-operative malnutrition in patients with ovarian cancer: what are the clinical implications? results of a prospective study. Cancers 16(3):622. 10.3390/cancers16030622 38339372 10.3390/cancers16030622 PMC10854561

[10] Stelten ST, Schofield CH, Hartman YV, Lopez PE, Kenter GE, Newton RO et al (2022) Association between energy balance-related factors and clinical outcomes in patients with ovarian cancer: a systematic review and meta-analysis. Cancers 14(19):4567. 10.3390/cancers14194567 36230490 10.3390/cancers14194567 PMC9559499

[11] Laky BR, Janda MO, Kondalsamy-Chennakesavan SR, Cleghorn GE, Obermair AN (2010) Pretreatment malnutrition and quality of life - association with prolonged length of hospital stay among patients with gynecological cancer: a cohort study. BMC Cancer 10(1):232. 10.1186/1471-2407-10-232 20497581 10.1186/1471-2407-10-232 PMC2894793

[12] Wood NI, Morton MO, Shah SH, Yao ME, Barnard HA, Tewari SU et al (2023) Association between CT-based body composition assessment and patient outcomes during neoadjuvant chemotherapy for epithelial ovarian cancer. Gynecol Oncol 169:55–63. 10.1016/j.ygyno.2022.11.024 36508759 10.1016/j.ygyno.2022.11.024

[13] Huang CY, Yang YC, Chen TC, Chen JR, Chen YJ, Wu MH et al (2020) Muscle loss during primary debulking surgery and chemotherapy predicts poor survival in advanced-stage ovarian cancer. J Cachexia Sarcopenia Muscle 11(2):534–546. 10.1002/jcsm.12524 31999069 10.1002/jcsm.12524 PMC7113537

[14] Cuello MA, Gómez FE, Wichmann IG, Suárez FE, Kato SU, Orlandini EL, et al (2023) Body composition and metabolic dysfunction really matter for the achievement of better outcomes in high-grade serous ovarian cancer. Cancers (Basel) 15(4) 10.3390/cancers15041156 10.3390/cancers15041156 PMC9953877 36831500

[15] Arends JA (2024) Malnutrition in cancer patients: causes, consequences and treatment options. Eur J Surg Oncol 50(5):107074. 10.1016/j.ejso.2023.107074 37783594 10.1016/j.ejso.2023.107074

[16] Balogun NY, Forbes AL, Widschwendter MA, Lanceley AN (2012) Noninvasive nutritional management of ovarian cancer patients: beyond intestinal obstruction. Int J Gynecol Cancer 22(6):1089–1095. 10.1097/IGC.0b013e318256e4d3 22688964 10.1097/IGC.0b013e318256e4d3

[17] Cederholm T, Jensen GL, Correia M, Gonzalez MC, Fukushima R, Higashiguchi T et al (2019) GLIM criteria for the diagnosis of malnutrition - a consensus report from the global clinical nutrition community. J Cachexia Sarcopenia Muscle 10(1):207–217. 10.1002/jcsm.12383 30920778 10.1002/jcsm.12383 PMC6438340

[18] Anjanappa MI, Corden MI, Green AN, Roberts DA, Hoskin PE, McWilliam AL et al (2020) Sarcopenia in cancer: risking more than muscle loss. Tech Innov Patient Support Radiat Oncol. 16:50–57. 10.1016/j.tipsro.2020.10.001 33385074 10.1016/j.tipsro.2020.10.001 PMC7769854

[19] Bauer JU, Morley JO, Schols AN, Ferrucci LU, Cruz-Jentoft AL, Dent EL et al (2019) Sarcopenia: a time for action. An SCWD Position Paper. J Cachexia Sarcopenia Muscle 10(5):956–961. 10.1002/jcsm.12483 31523937 10.1002/jcsm.12483 PMC6818450

[20] Kiss NI, Loeliger JE, Findlay ME, Isenring EL, Baguley BR, Boltong AN et al (2020) Clinical Oncology Society of Australia: position statement on cancer-related malnutrition and sarcopenia. Nutr Diet 77(4):416–425. 10.1111/1747-0080.12631 32803904 10.1111/1747-0080.12631 PMC7540290

[21] Prado CA, Landi FR, Chew SA, Atherton PH, Molinger JE, Ruck TO et al (2022) Advances in muscle health and nutrition: a toolkit for healthcare professionals. Clin Nutr 41(10):2244–2263. 10.1016/j.clnu.2022.07.041 36081299 10.1016/j.clnu.2022.07.041

[22] Aprile GI, Basile DE, Giaretta RE, Schiavo GE, La Verde NI, Corradi ET, et al. (2021) The clinical value of nutritional care before and during active cancer treatment. Nutrients 13(4); 10.3390/nu13041196 10.3390/nu13041196 PMC8065908 33916385

[23] Arends J, Bachmann P, Baracos V, Barthelemy N, Bertz H, Bozzetti F et al (2017) ESPEN guidelines on nutrition in cancer patients. Clin Nutr 36(1):11–48. 10.1016/j.clnu.2016.07.015 27637832 10.1016/j.clnu.2016.07.015

[24] Samaranayake SH, Barker DA, Windsor AP (2023) Optimal cancer care pathways – the ideal versus reality for patient-centric cancer care during COVID-19. Aust Health Rev 47(4):472–479. 10.1071/AH23060 37369140 10.1071/AH23060

[25] Croisier E, Morrissy A, Brown T, Grigg A, Chan P, Goh J et al (2022) Nutrition risk screening and implications for patients with gynaecological cancers undergoing pelvic radiotherapy and/or other treatment modalities: a retrospective observational study. Nutr Diet 79(2):217–228. 10.1111/1747-0080.12712 34854202 10.1111/1747-0080.12712

[26] Johnston EL, Ibiebele TO, van der Pols JO, Webb PE, Group tOS (2021) Dietitian encounters after treatment for ovarian cancer. J Hum Nutr Diet 34(6):1053–63. 10.1111/jhn.12898 10.1111/jhn.12898 33749900

[27] Johnston EA, Ekberg S, Jennings B, Jagasia N, van der Pols JC. Discussing diet, nutrition, and body weight after treatment for gynecological cancer: a conversation analytic study of outpatient consultations. J Cancer Surviv. 2023 10.1007/s11764-023-01345-w 10.1007/s11764-023-01345-w PMC11081991 36897546

[28] Eysenbach GU (2004) Improving the quality of Web surveys: the Checklist for Reporting Results of Internet E-Surveys (CHERRIES). J Med Internet Res 6(3):e34. 10.2196/jmir.6.3.e34 15471760 10.2196/jmir.6.3.e34 PMC1550605

[29] Kiss NI, Bauer JU, Boltong AN, Brown TE, Isenring LI, Loeliger JE et al (2020) Awareness, perceptions and practices regarding cancer-related malnutrition and sarcopenia: a survey of cancer clinicians. Support Care Cancer 28(11):5263–5270. 10.1007/s00520-020-05371-7 32103357 10.1007/s00520-020-05371-7

[30] Deftereos IR, Kiss NI, Brown TE, Carey SH, Carter VA, Usatoff VA et al (2021) Awareness and perceptions of nutrition support in upper gastrointestinal cancer surgery: a national survey of multidisciplinary clinicians. Clin Nutr ESPEN 46:343–349. 10.1016/j.clnesp.2021.09.734 34857218 10.1016/j.clnesp.2021.09.734

[31] Laing E, Kiss N, Krishnasamy M, Gough K, Michael M (2022) Exploring health professional knowledge and management of nutritional complications in neuroendocrine cancer patients: results of an international multidisciplinary survey. Clin Nutr ESPEN 49:466–473. 10.1016/j.clnesp.2022.02.124 35623853 10.1016/j.clnesp.2022.02.124

[32] Hustad KS, Koteng LH, Urrizola A, Arends J, Bye A, Dajani O, et al (2025) Practical cancer nutrition, from guidelines to clinical practice: a digital solution to patient-centred care. ESMO Open. 10(4) 10.1016/j.esmoop.2025.104529 10.1016/j.esmoop.2025.104529 PMC11998113 40179818

[33] Viana ED, Oliveira IS, Rechinelli AN, Marques IS, Souza VA, Spexoto MA et al (2020) Malnutrition and nutrition impact symptoms (NIS) in surgical patients with cancer. PLoS ONE 15(12):e0241305. 10.1371/journal.pone.0241305 33320857 10.1371/journal.pone.0241305 PMC7737886

[34] Prado CA, Laviano AL, Gillis CH, Sung AN, Gardner MA, Yalcin SU et al (2022) Examining guidelines and new evidence in oncology nutrition: a position paper on gaps and opportunities in multimodal approaches to improve patient care. Support Care Cancer 30(4):3073–3083. 10.1007/s00520-021-06661-4 34811570 10.1007/s00520-021-06661-4 PMC8857008

[35] Omlin AU, Blum DA, Wierecky JA, Haile SA, Ottery FA, Strasser FL (2013) Nutrition impact symptoms in advanced cancer patients: frequency and specific interventions, a case-control study. J Cachexia Sarcopenia Muscle 4(1):55–61. 10.1007/s13539-012-0099-x 23307589 10.1007/s13539-012-0099-x PMC3581613

[36] Johnston EA, Ibiebele TI, Friedlander ML, Grant PT, van der Pols JC, Webb PM et al (2023) Association of protein intake with recurrence and survival following primary treatment of ovarian cancer. Am J Clin Nutr 118(1):50–58. 10.1016/j.ajcnut.2023.05.002 37146759 10.1016/j.ajcnut.2023.05.002

[37] Johnston EA, Ekberg S, Jennings B, Jagasia N, van der Pols JC, Webb PM (2022) Dietary practices after primary treatment for ovarian cancer: a qualitative analysis from the OPAL study. J Acad Nutr Diet 122(9):1607–28.e12. 10.1016/j.jand.2022.05.014 35595188 10.1016/j.jand.2022.05.014

[38] Marshall KA, Loeliger JE, Nolte LI, Kelaart AM, Kiss NI (2019) Prevalence of malnutrition and impact on clinical outcomes in cancer services: a comparison of two time points. Clin Nutr 38(2):644–651. 10.1016/j.clnu.2018.04.007 29789167 10.1016/j.clnu.2018.04.007

[39] Muscaritoli MA, Bar-Sela GI, Battisti NI, Belev BO, Contreras-Martínez JO, Cortesi EN, et al (2023) Oncology-led early identification of nutritional risk: a pragmatic, evidence-based protocol (PRONTO). Cancers (Basel) 15(2). 10.3390/cancers15020380 10.3390/cancers15020380 PMC9856655 36672329

[40] Baracos VE, Arribas L (2018) Sarcopenic obesity: hidden muscle wasting and its impact for survival and complications of cancer therapy. Annals Oncol 29:ii1–ii9. 10.1093/annonc/mdx810 10.1093/annonc/mdx810 29506228

[41] Prado CA, Wells JCK, Smith SR, Stephan BCM, Siervo M (2012) Sarcopenic obesity: a Critical appraisal of the current evidence. Clin Nutr 31(5):583–601. 10.1016/j.clnu.2012.06.010 22809635 10.1016/j.clnu.2012.06.010

[42] Momenimovahed ZO, Tiznobaik AZ, Taheri SA, Salehiniya HA (2019) Ovarian cancer in the world: epidemiology and risk factors. Int J Womens Health. 11:287–299. 10.2147/ijwh.S197604 31118829 10.2147/IJWH.S197604 PMC6500433

[43] Grammatikopoulou MG, Nigdelis MP, Goulis DG (2022) Weight gain in midlife women: understanding drivers and underlying mechanisms. Current Opinion in Endocrine and Metabolic Research. 27:100406. 10.1016/j.coemr.2022.100406

[44] Cancer Council Victoria and Department of Health Victoria (2021) Optimal care pathway for women with ovarian cancer, 2nd edn. Cancer Council Victoria, Melbourne. Available from: https://www.cancer.org.au/assets/pdf/ovarian-cancer-optimal-cancer-care-pathway. Accessed 25 May 2025

[45] Pati S, Irfan W, Jameel A, Ahmed S, Shahid RK (2023) Obesity and cancer: a current overview of epidemiology, pathogenesis, outcomes, and management. Cancers (Basel). 15(2). 10.3390/cancers15020485 10.3390/cancers15020485 PMC9857053 36672434

[46] Muscaritoli MA, Corsaro EM, Molfino AL (2021) Awareness of cancer-related malnutrition and its management: analysis of the results from a survey conducted among medical oncologists. Front Oncol 11:682999. 10.3389/fonc.2021.682999 34055649 10.3389/fonc.2021.682999 PMC8155516

[47] Loeliger JE, Edbrooke LA, Daly RO, Stewart JA, Bucci LU, Puskas CA, et al (2022) Development and feasibility of an inpatient cancer-related sarcopenia pathway at a major cancer centre. Int J Environ Res Public Health 19(7). 10.3390/ijerph19074038 10.3390/ijerph19074038 PMC8997788 35409719

[48] De Felice FR, Malerba SI, Nardone VA, Salvestrini VI, Calomino NA, Testini MA et al (2025) Progress and challenges in integrating nutritional care into oncology practice: results from a national survey on behalf of the NutriOnc research group. Nutrients 17(1):188. 10.3390/nu17010188 39796623 10.3390/nu17010188 PMC11722632

