# Supplementary material for: Exploring awareness, perceptions, and practices relating to nutritional status and low muscle mass in patients with ovarian cancer
Source: Support Care Cancer. 2025 Jul 21;33(8):709. doi: 10.1007/s00520-025-09739-5 (PMC12279610; doi:10.1007/s00520-025-09739-5)
Supplement: Supplementary file 1 — Supplementary file1 (DOCX 33 KB) [file 520_2025_9739_MOESM1_ESM.docx]

**Article title:** Exploring awareness, perceptions and practices relating to nutritional status and low muscle mass in patients with ovarian cancer

**Journal name**: Supportive Care in Cancer

**Authors:** Benna-Doyle, S., Kiss, N., Laing, E., Loeliger, J., Baguley, B.J.

**Corresponding author:** Sarah Benna-Doyle

**Affiliations:**

Institute for Physical Activity and Nutrition, Deakin University, Geelong, VIC, Australia

School of Exercise and Nutrition Sciences, Deakin University, Burwood, VIC, Australia

Nutrition and Speech Pathology Department, Peter MacCallum Cancer Centre, Melbourne, VIC, Australia

**Email:** sbennadoyle@deakin.edu.au

**Online Resource 1**: Survey Instrument

**Section 1: Demographic questions**

In this first section, we would like to ask you a few questions about your profession and work environment:

1. What is your professional discipline?

- Medical Oncologist
- Radiation oncologist
- Surgeon
- Other medical specialist (please specify)
- Nurse
- Dietitian
- Exercise physiologist
- Physiotherapist
- Other allied health (please specify)
- Other health professional (please specify)

2. In which state or territory do you currently work?

- Australian Capital Territory
- New South Wales
- Northern Territory
- Queensland
- South Australia
- Tasmania
- Victoria
- Western Australia

3. In what practice setting do you primarily work? (you may select more than one response if you split your time between practice settings)

- Public hospital
- Private hospital
- Primary health care
- Community health care
- Private practice
- Other (please specify)

4. Which geographic setting best represents your work location?

- Metropolitan
- Rural/regional
- Both metropolitan and rural/regional

5. How long have you been working with, treating, or managing patients with ovarian cancer (OC)?

- < 1 year
- 1-5 years
- 6-10 years
- 11- 20 years
- >20 years

6. What proportion of your time would you estimate is spent working with, treating, or managing patients with OC?

- ≥75%, all or almost all of your caseload
- ≥ 50% but <75%, at least half or slightly more of your case load
- ≥25% but <50%, some of your caseload
- ≥5% but <25%, a little of your caseload
- <5% of your caseload

**Section 2. Awareness and perspectives of nutrition-related issues in OC**

Next, we would like to ask you a series of questions regarding your experience with nutrition-related issues and your perspective of these issues in practice:

7. In your experience, how often do patients with OC report the following nutrition-related symptoms? Please rate how often patients report experiencing the below symptoms. If there are any symptoms of importance that have not been included on this list, please select ‘other’ and write them.

| **Symptom** | **Never** | **Sometimes** | **About half the time** | **Most of the time** | **Always** |
| --- | --- | --- | --- | --- | --- |
| Abdominal pain/cramping |  |  |  |  |  |
| Bloating |  |  |  |  |  |
| Wind/gas |  |  |  |  |  |
| Indigestion/heartburn |  |  |  |  |  |
| Early satiety |  |  |  |  |  |
| Anorexia or decreased appetite |  |  |  |  |  |
| Nausea |  |  |  |  |  |
| Vomiting |  |  |  |  |  |
| Diarrhoea |  |  |  |  |  |
| Constipation |  |  |  |  |  |
| Changes to taste/smell |  |  |  |  |  |
| Fatigue |  |  |  |  |  |
| Other, please specify: |  | | | | |

8. In your experience, when are patients most likely to experience the greatest symptom burden? (please select all that apply)

- At diagnosis
- After completion of surgery
- During chemotherapy
- After chemotherapy
- During radiotherapy
- After completion of radiotherapy
- During immunotherapy
- After completion of immunotherapy
- All of the above
- Other, please specify_______

9. Do you observe any of the following nutrition-related issues among your patients with OC, and if so, at what time-point do to they tend to occur? (Select all that apply)

| **Nutrition-related issue** | **At time of diagnosis** | **During treatment** | **Post**  **Treatment** | **I do not observe this nutrition related issue** |
| --- | --- | --- | --- | --- |
| Unintentional weight loss |  |  |  |  |
| Intentional weight loss |  |  |  |  |
| Overweight or obesity |  |  |  |  |
| Weight gain |  |  |  |  |
| Sarcopenia (muscle loss) |  |  |  |  |
| Self-initiated dietary changes |  |  |  |  |

10. In your experience, which of the following symptoms are most likely to lead to the development of nutrition-related issues (such as reduced food intake, deterioration of nutritional status, weight and/or muscle loss?) in patients with OC?
Please rate the below symptoms on a scale of 0-7. 0- is extremely unlikely and 7-is extremely likely

| **Symptom** | **0-extremely unlikely** | **2** | **3** | **4** | **5** | **6** | **7-Extremely Likely** |
| --- | --- | --- | --- | --- | --- | --- | --- |
| Anorexia or decreased appetite |  |  |  |  |  |  |  |
| Changes to taste/smell |  |  |  |  |  |  |  |
| Early satiety |  |  |  |  |  |  |  |
| Nausea |  |  |  |  |  |  |  |
| Vomiting |  |  |  |  |  |  |  |
| Diarrhoea |  |  |  |  |  |  |  |
| Constipation |  |  |  |  |  |  |  |
| Abdominal pain |  |  |  |  |  |  |  |
| Fatigue |  |  |  |  |  |  |  |
| Other, (please specify): |  | | | | | | |

11. In your experience, please indicate the treatments that are most likely to lead to nutrition-related issues (e.g. reduced food intake, weight loss)? (Select all that apply)

- Chemotherapy
- Surgery
- Radiotherapy
- Immunotherapy
- Other, please specify_______
- I’m not sure
- I don’t find treatment leads to nutrition-related issues in my patients with OC

**Section 3. Understanding and perspectives of malnutrition and sarcopenia**

In this section we would like to ask you two questions related to the importance of malnutrition and sarcopenia (low muscle mass) in OC:

12. In your experience, how important is nutritional status (i.e. if a patient is malnourished or well nourished) to the overall treatment and management of patients with OC? Please rate importance of nutritional status on a scale of 0-7. 0- is not at all important and 7-is extremely important

| **0-Not at all important** | **2** | **3** | **4** | **5** | **6** | **7-Extremely important** |
| --- | --- | --- | --- | --- | --- | --- |

13. In your experience, how important is sarcopenia (i.e., low muscle mass) to the overall treatment and management of patients with OC? Please rate importance of sarcopenia on a scale of 0-7. 0- is not at all important and 7-is extremely important

| **0-Not at all important** | **2** | **3** | **4** | **5** | **6** | **7-Extremely important** |
| --- | --- | --- | --- | --- | --- | --- |

**Section 4. Screening and referral practices**

In the next section we would like to ask you a series of questions regarding nutrition risk screening and referral practices for patients with OC in your health services.

14. In your health service who is responsible for nutrition risk screening for patients with OC?

- Medical Oncologist
- Radiation oncologist
- Surgeon
- Other medical specialist (please specify)
- Nurse
- Dietitian
- Exercise physiologist
- Physiotherapist
- Other allied health (please specify)______
- Other health professional (please specify)_____
- I’m not sure
- To my knowledge my health service does not screen for nutrition risk

Branching logic (skip Q15 if “I’m not sure” or “to my knowledge my health service does not screen for nutrition risk” is selected in Q14)

15. Which, if any, of the following tools are used for nutrition risk screening in your health service? select all that apply)

- Malnutrition Screening Tool (MST)
- Malnutrition Screening Tool for Cancer Patients (MSTC)
- The Malnutrition Universal Screening Tool (MUST)
- The Nutritional Risk Screening Tool (NRS-2002)
- Patient-Generated Subjective Global Assessment Short Form (PG-SGA SF)
- Biochemical markers albumin or prealbumin
- Other, please specify_______
- I’m not sure

Branching logic (skip Q16 if “I’m not sure” or “to my knowledge my health service does not screen for nutrition risk” is selected in Q14)

16. In your experience, at what time point does nutrition risk screening usually occur? (Select all that apply)

- At diagnosis
- Before surgery
- After surgery
- Before chemotherapy
- During chemotherapy
- After chemotherapy
- Before radiotherapy
- During radiotherapy
- After radiotherapy
- Before immunotherapy
- During immunotherapy
- After immunotherapy
- Initial attendance to outpatient
- On admission as inpatient
- Other (please specify)______
- I’m not sure

17. In your health service who is responsible for sarcopenia (low muscle mass) risk screening for patients with OC? (select all that apply)

- Medical Oncologist
- Radiation oncologist
- Surgeon
- Other medical specialist (please specify)
- Nurse
- Dietitian
- Exercise physiologist
- Physiotherapist
- Other allied health (please specify)
- Other health professional (please specify)
- I’m not sure
- To my knowledge my health service does not screen for sarcopenia (low muscle mass)

Branching logic (skip Q18 if “I’m not sure” or “to my knowledge my health service does not screen...” is selected in Q17)

18. Do you or your team member use any of the below tools or methods to screen or assess for sarcopenia? (Select all that apply)

- SARC-F
- SARC-F in combination with calf circumference
- Body Mass Index (BMI)
- Bioelectric impedance analysis (BIA)
- Dual energy X-ray (DXA)
- Computed Tomography (CT) scan analysis
- A measure of muscle strength e.g., hand grip strength (HGS)
- A measure of muscle function e.g., gait speed
- Other, please specify_______
- I’m not sure

Branching logic (skip Q19 if “I’m not sure” or “to my knowledge my health service does not screen...” is selected in Q17)

19. At what time point does screening or assessment of sarcopenia (low muscle mass) usually occur?

(Select all that apply)

- At diagnosis
- Before surgery
- After surgery
- Before chemotherapy
- During chemotherapy
- After chemotherapy
- Before radiotherapy
- During radiotherapy
- After radiotherapy
- Before immunotherapy
- During immunotherapy
- After immunotherapy
- Initial attendance to outpatient
- On admission as inpatient
- Other (please specify)______
- I’m not sure

20. In your health service, are dietetic services available for you to refer patients?

- Yes
- No
- I’m not sure

Branching logic: (Q21 only if yes to Q20)

21. In your experience, what are the primary reason(s) that patients with OC would require a dietitian referral?? (Select all that apply)

- Unintentional weight loss or malnutrition
- Sarcopenia (muscle loss)
- Weight management advice due to overweight or obesity
- Management of symptoms or treatment side effects, please specify_____
- Patient or carer request
- Dietetic referrals are made automatically as part of treatment-specific pathways. (Please specify for what treatments)
- Other, please specify_____

Branching logic: (Q22 only if “dietetic referrals are made automatically as part of treatment-specific pathways” is selected in Q21.)

22. Could you please briefly describe the criteria for an automatic referral to a dietitian in your health service?

Branching logic: (Q23 only if yes to Q20)

23. In your experience, at what time-point(s) is it most common for patients with OC to be referred to a dietitian? (Select all that apply)

- At diagnosis, before any treatment
- During neoadjuvant chemotherapy
- After neoadjuvant chemotherapy before surgery
- After surgery before adjuvant chemotherapy
- During adjuvant chemotherapy
- After adjuvant chemotherapy
- Before radiotherapy
- During radiotherapy
- After radiotherapy
- Before immunotherapy
- During immunotherapy
- After immunotherapy
- Other, please specify

**Section 5. Barriers**

In the next section we would like to ask two final questions regarding what you perceive to be barriers to the provision of nutrition-related services for patients with OC in your health services.

24. What do you consider to be barriers to providing nutrition-related services to patients with OC in your health service?

| **Potential barrier in your health service** | Strongly disagree | Somewhat disagree | Neither agree nor disagree | Somewhat agree | Strongly agree |
| --- | --- | --- | --- | --- | --- |
| Patients with OC are not prioritised for nutrition services |  |  |  |  |  |
| The medical team do not view nutrition as a priority for these patients. |  |  |  |  |  |
| The medical team do not view sarcopenia (muscle loss) as a priority concern for these patients. |  |  |  |  |  |
| There is no time to screen patients with OC for nutrition risk |  |  |  |  |  |
| There are no established processes to screen patients with OC for nutrition risk |  |  |  |  |  |
| There is no time to screen patients with OC for sarcopenia |  |  |  |  |  |
| There are no established processes to screen patients with OC for sarcopenia |  |  |  |  |  |
| There are limited dietitian services available |  |  |  |  |  |
| There is a lack of evidence-based guidelines specific to OC to guide nutrition-related practices |  |  |  |  |  |
| There is a lack of standard referral or clinical pathways for nutrition services for patients with OC |  |  |  |  |  |
| There aren’t any barriers to providing nutrition-related services to patients |  |  |  |  |  |
| Other, please specify: |  | | | | |
