# Supplementary material for: Exploring awareness, perceptions, and practices relating to nutritional status and low muscle mass in patients with ovarian cancer
Source: Support Care Cancer. 2025 Jul 21;33(8):709. doi: 10.1007/s00520-025-09739-5 (PMC12279610; doi:10.1007/s00520-025-09739-5)
Supplement: Supplementary file 2 — Supplementary file2 (DOCX 23 KB) [file 520_2025_9739_MOESM2_ESM.docx]

**Article title:** Exploring awareness, perceptions and practices relating to nutritional status and low muscle mass in patients with ovarian cancer

**Journal name**: Supportive Care in Cancer

**Authors:** Benna-Doyle, S., Kiss, N., Laing, E., Loeliger, J., Baguley, B.J.

**Corresponding author:** Sarah Benna-Doyle

**Affiliations:**

Institute for Physical Activity and Nutrition, Deakin University, Geelong, VIC, Australia

School of Exercise and Nutrition Sciences, Deakin University, Burwood, VIC, Australia

Nutrition and Speech Pathology Department, Peter MacCallum Cancer Centre, Melbourne, VIC, Australia

**Email:** sbennadoyle@deakin.edu.au

**Supplementary Table 1**: Healthcare professional (N= 57) reported time points where patients experience the greatest symptom burden, nutrition and sarcopenia risk screening occur and dietetic referrals are made

|  | **Symptom burden** | **Nutrition risk screening** | **Sarcopenia screening** | **Dietetic referral** |
| --- | --- | --- | --- | --- |
| Diagnosis | 32 (56%) | 10 (18%) | 7 (12%) | 21 (37%) |
| Hospital admission | - | 15 (26%) | 2 (4%) | - |
| Outpatient attendance | - | 9 (16%) | 2 (4%) | - |
| *Surgery* |  |  |  |  |
| Pre-surgery | - | 14 (25%) | 8 (14%) | - |
| Post-surgery | 12 (21%) | 10 (18%) | 8 (14%) | 18 (32%) |
| *Chemotherapy* ^a^ |  |  |  |  |
| Pre-chemotherapy | - | 12 (21%) | 4 (7%) | 18 (32%) |
| During chemotherapy | 30 (53%) | 9 (16%) | 5 (9%) | 31 (54%) |
| Post-chemotherapy | 8 (14%) | 5 (87%) | 6 (11%) | 18 (32%) |
| *Radiotherapy* |  |  |  |  |
| Pre-radiotherapy | - | 8 (14%) | 2 (4%) | 3 (5%) |
| During radiotherapy | 12 (21%) | 7 (12%) | 4 (7%) | 9 (16%) |
| Post-radiotherapy | 13 (23%) | 4 (7%) | 2 (4%) | 2 (4%) |
| *Immunotherapy* |  |  |  |  |
| Pre-immunotherapy | - | 3 (5%) | 1 (2%) | 1 (2%) |
| During immunotherapy | 9 (16%) | 4 (7%) | 4 (7%) | 2 (4%) |
| Post-immunotherapy | 3 (5%) | 3 (5%) | 3 (5%) | - |
| *Additional responses* |  |  |  |  |
| Not sure | - | 5 (9%) | 5 (9%) | - |
| Other | - | 2 (4%) | 4 (7%) | 6 (11%) |
| All the above | 10 (18%) | - | - | - |

Multiple responses were allowed

^a^ Dietetic referral captures both neoadjuvant and adjuvant chemotherapy
